# ChemGenXplore: an interactive tool for exploring and analysing chemical genomic data

**DOI:** 10.1093/bioinformatics/btag021

**Published:** 2026-01-13

**Authors:** Huda Ahmad, Hannah M Doherty, Sam T Benedict, James R J Haycocks, Ge Zhou, Patrick J Moynihan, Danesh Moradigaravand, Manuel Banzhaf

**Affiliations:** Institute of Microbiology and Infection and School of Biosciences, University of Birmingham, Birmingham, B15 2TT, United Kingdom; Institute of Microbiology and Infection and School of Biosciences, University of Birmingham, Birmingham, B15 2TT, United Kingdom; Newcastle University Biosciences Institute, Faculty of Medical Sciences, Newcastle University, Newcastle upon Tyne, NE2 4HH, United Kingdom; Newcastle University Biosciences Institute, Faculty of Medical Sciences, Newcastle University, Newcastle upon Tyne, NE2 4HH, United Kingdom; Laboratory of Infectious Disease Epidemiology, KAUST Center of Excellence for Smart Health and Biological and Environmental Science and Engineering (BESE) Division, King Abdullah University of Science and Technology (KAUST), Thuwal 23955-6900, Kingdom of Saudi Arabia; Institute of Microbiology and Infection and School of Biosciences, University of Birmingham, Birmingham, B15 2TT, United Kingdom; Laboratory of Infectious Disease Epidemiology, KAUST Center of Excellence for Smart Health and Biological and Environmental Science and Engineering (BESE) Division, King Abdullah University of Science and Technology (KAUST), Thuwal 23955-6900, Kingdom of Saudi Arabia; Institute of Microbiology and Infection and School of Biosciences, University of Birmingham, Birmingham, B15 2TT, United Kingdom; Newcastle University Biosciences Institute, Faculty of Medical Sciences, Newcastle University, Newcastle upon Tyne, NE2 4HH, United Kingdom

## Abstract

**Motivation:**

Chemical genomics is a powerful high-throughput approach to systematically link phenotypes to genotypes. However, the vast datasets generated remain challenging to explore due to the lack of integrated, interactive tools for visualization and analysis. Existing workflows often require multiple independent software tools, limiting data accessibility and collaboration. Therefore, we created a user-friendly platform that enables efficient exploration and sharing of chemical genomics data.

**Results:**

We developed ChemGenXplore, a web-based Shiny application designed to streamline the visualization and analysis of chemical genomic screens. It offers two primary functionalities: one for exploring pre-implemented datasets and another for analysing user-uploaded datasets. ChemGenXplore enables users to visualize phenotypic profiles, assess gene–gene and condition–condition correlations, perform GO and KEGG enrichment analysis, and generate customizable, interactive heatmaps. To further support collaborative research, ChemGenXplore also facilitates the comparative analysis of chemical genomic and other omics datasets. By consolidating these features into a single interactive and accessible tool, ChemGenXplore facilitates data sharing, enhances reproducibility, and promotes collaboration within the research community.

**Availability and implementation:**

ChemGenXplore is freely accessible as a web application at https://chemgenxplore.kaust.edu.sa/. Source code and documentation, including instructions for local installation, are provided on GitHub (https://github.com/Hudaahmadd/ChemGenXplore). A Docker image is also available on DockerHub (https://hub.docker.com/r/hudaahmad/chemgenxplore) to ensure reproducibility and simplify installation.

## 1 Introduction

Over recent decades, advances in high-throughput technologies have facilitated the generation of large-scale molecular and phenotypic data across a wide range of microbial species. These advancements have led to gene expression studies across thousands of conditions (Brown *et al.* 2014, Gerstein *et al.* 2014, Tsankov *et al.* 2015, Chen *et al.* 2018), as well as genome-wide phenotypic profiling discovering new biology (Tong *et al.* 2001, Schuldiner *et al.* 2005, Gomez Maria and Neyfakh Alexander 2006, Fajardo *et al.* 2008, Tamae *et al.* 2008, Typas *et al.* 2010, Nichols *et al.* 2011, Arend *et al.* 2016, Koo *et al.* 2017, Bobonis *et al.* 2022). However, the rapid accumulation and complexity of high-throughput biological data present significant computational challenges, necessitating the development of efficient tools for data integration, processing, and interpretation (Houle *et al.* 2010, Schadt *et al.* 2010, Sullivan *et al.* 2014). As high-throughput approaches continue to expand, addressing these challenges is essential for translating large-scale biological data into knowledge.

Chemical genomics is a high-throughput screening method that facilitates the functional annotation of orphan genes by systematically evaluating the effect of gene disruption on fitness across diverse chemical and environmental perturbations (Cain *et al.* 2020). This approach generates phenotypic profiles that serve as a valuable resource for future investigations into gene function and cellular responses (Schuldiner *et al.* 2005, Nichols *et al.* 2011, Cain *et al.* 2020, Doherty *et al.* 2023, Alodaini *et al.* 2024, Hayward *et al.* 2025). Such studies have contributed to the construction of genotype–phenotype relationships and gene interaction networks (Tong *et al.* 2001, Schuldiner *et al.* 2005, Nichols *et al.* 2011, Koo *et al.* 2017). These experiments involve thousands of gene knockouts, generated through methods such as transposon insertion sequencing (Tn-seq) or deletion libraries, and each mutant is tested across hundreds of stress conditions (Nichols *et al.* 2011, Koo *et al.* 2017, Kritikos *et al.* 2017, Williams *et al.* 2025). Such datasets cannot be manually analysed to fully capture the phenotypic profile of an organism. In response to these large-scale datasets, a variety of advanced analysis approaches have emerged (Collins *et al.* 2006, Dixon *et al.* 2009, Nichols *et al.* 2011, French *et al.* 2016, Shiver *et al.* 2017, Doherty *et al.* 2023). These approaches provide systematic methods to analyse and quantify phenotypic changes, generating scored datasets. However, the key challenge lies in extracting meaningful biological insights from these scores. Many current pipelines rely on multiple independent tools, such as Cluster 3.0 (de Hoon *et al.* 2004) for hierarchical clustering, Java TreeView (Saldanha 2004) for data visualization, in-house scripts, and manual analysis to understand phenotypic profiles. These tools require extensive preprocessing, manual parameter selection, and frequent transitions between platforms, making data-exploration inefficient and time-consuming. Furthermore, the fragmented nature of existing resources limits the accessibility and reproducibility of chemical genomics data, posing barriers to collaborative research.

To address these challenges and facilitate more accessible exploration of chemical genomics data, we introduce ChemGenXplore. ChemGenXplore is a web-based, interactive platform that enables users to visualize phenotypic data, compute gene–gene and condition–condition correlations, perform Gene Ontology (GO) and Kyoto Encyclopedia of Genes and Genomes (KEGG) enrichment analyses, and generate customizable interactive heatmaps with clustering options for genes and conditions. It also supports comparative integration of chemical genomic datasets with other omics data, enabling users to identify and compare patterns across datasets. Additionally, ChemGenXplore serves as a user-friendly tool for sharing chemical genomics resources, enhancing accessibility, reproducibility, and collaboration within the field.

## 2 Materials and methods

### 2.1 Overview

**Figure 1:**
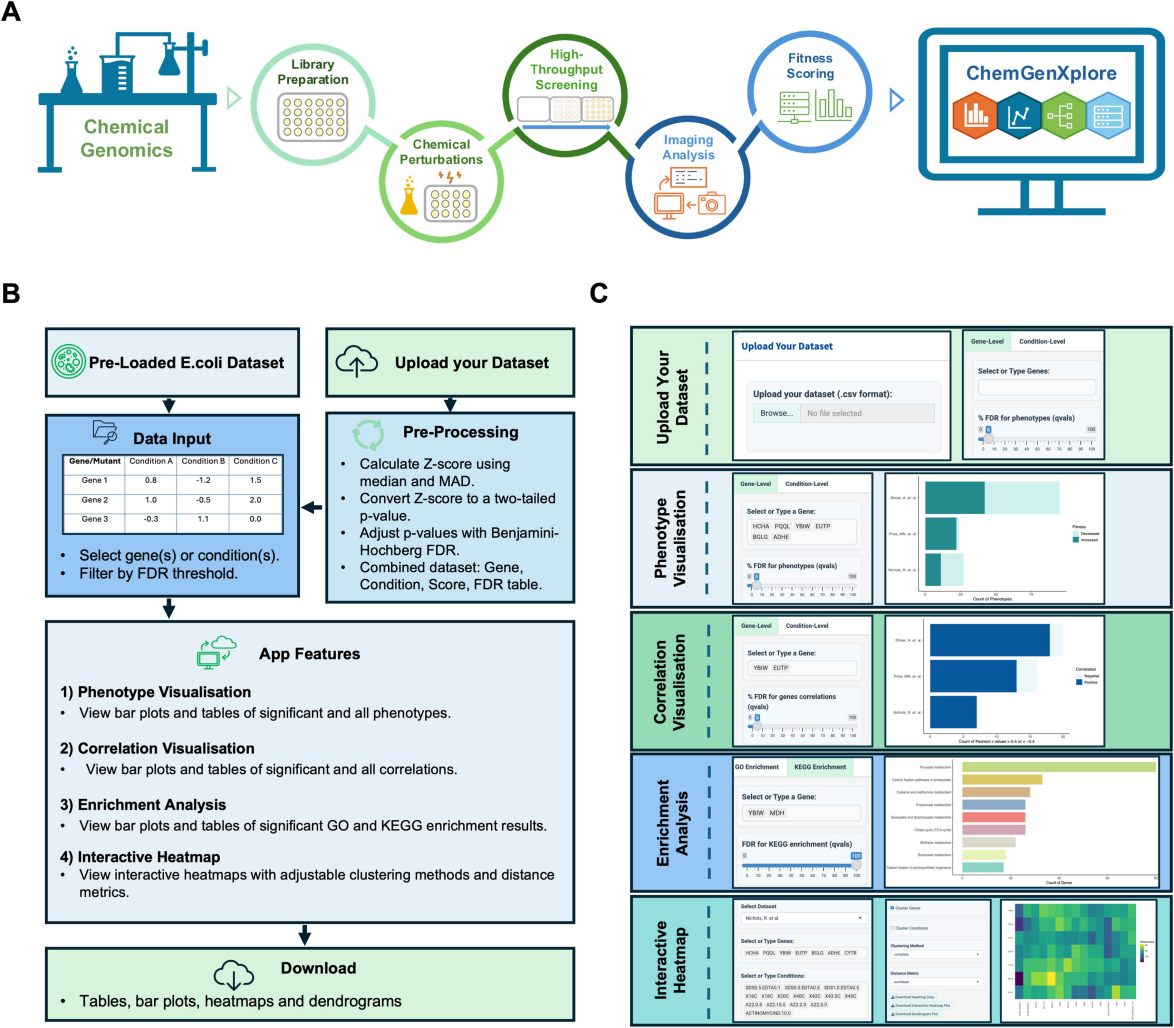
General workflow of chemical genomic screening. (A) Mutant or microbial strain libraries are arrayed onto plates with chemical perturbations, followed by high-throughput screening, image analysis, and fitness scoring. ChemGenXplore is then used for data visualization and exploration of the scored dataset. (B) In ChemGenXplore users select gene(s) and/or condition(s) of interest to explore phenotypic scores via bar plots and data tables. Other features include gene–gene or/and condition-condition correlation analysis, GO and KEGG enrichment, and interactive heatmaps. Users can also upload their own datasets and export all visualizations and results. (C) ChemGenXplore outputs examples from ChemGenXplore: interface panels and plots from each feature.

ChemGenXplore is designed to facilitate the exploration and interpretation of high-throughput chemical genomic screens. Figure 1 provides a schematic overview of the chemical genomic screening workflow and the role of ChemGenXplore in the data analysis pipeline. In a typical screen, mutant or microbial strain libraries are arrayed onto agar media containing specific chemical stressors. Following incubation, a phenotype such as colony growth is measured through high-throughput imaging, and image analysis pipelines are used to quantify strain fitness. Once the fitness scores are computed, ChemGenXplore enables users to interactively visualize, filter, and interpret the dataset, supporting downstream analyses such as phenotype profiling, correlation mapping, enrichment analysis, and heatmap clustering. By integrating these features into a single interface, ChemGenXplore streamlines the interpretation of complex chemical genomic data and enhances biological insight into gene- and condition-specific phenotypes.

### 2.2 Data input

**Figure 2:**
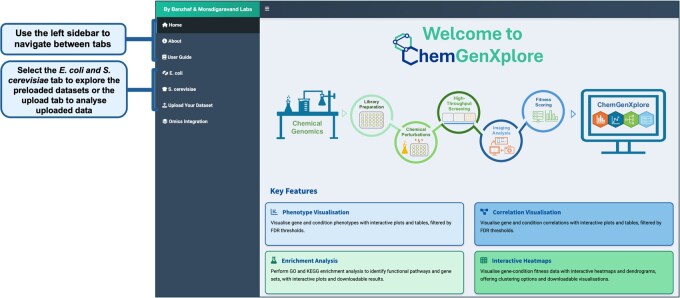
Data input. ChemGenXplore supports both pre-implemented *E. coli* and *S. cerevisiae* datasets and user-uploaded datasets.

ChemGenXplore allows the upload of multiple datasets, with upload size up to 300 MB to accommodate the large-scale nature of chemical genomic screens. The current implementation includes three pre-integrated, publicly available chemical genomic studies from *Escherichia coli* (Nichols *et al.* 2011, Shiver *et al.* 2017, Price *et al.* 2018) and one *Saccharomyces cerevisiae* dataset (Viéitez *et al.* 2022) (Fig. 2). These datasets provide fitness scores under a wide range of conditions, forming the foundation for the analyses performed in ChemGenXplore. Uploaded datasets must be provided as CSV files containing a single header row. The first column should include unique gene or strain identifiers, which are interpreted as row names. All subsequent columns must contain numeric fitness measurements for individual conditions, with each condition represented by a unique column header. Missing values are permitted and are processed using pairwise-complete methods. Any non-numeric entry in the measurement columns will result in an error.

### 2.3 ChemGenXplore web application

ChemGenXplore was developed as an interactive Shiny application using R (https://www.r-project.org/). The web application is freely accessible at https://chemgenxplore.kaust.edu.sa/. The full source code is publicly available at https://github.com/Hudaahmadd/ChemGenXplore. A pre-configured Docker image is provided on DockerHub (https://hub.docker.com/r/hudaahmad/chemgenxplore) to enable reproducible, dependency-free deployment.

### 2.4 Statistical analysis

*P*-values for fitness scores are derived from robust *Z*-scores. Correlation *P*-values are calculated from Pearson correlation coefficients using the *t* distribution. All *P*-values are adjusted using the Benjamini–Hochberg false discovery rate (FDR). Missing values are allowed and handled via pairwise-complete observations. For user-uploaded datasets, fitness-score FDRs and all correlations are computed on the full matrix prior to any filtering, and *P*-values are FDR-adjusted across all tested pairs. Distances for clustering are computed using pairwise-complete observations, requiring at least two-thirds shared measurements between pairs.

### 2.5 Clustering and enrichment analyses

Hierarchical clustering is performed in R (hclust) with user-selectable linkage methods. Distances are computed using pairwise-complete observations with standard numeric or correlation-based measures. Heatmaps and dendrograms are generated using ComplexHeatmap (https://bioconductor.org/packages/release/bioc/html/ComplexHeatmap.html) (Gu 2022). Clustering within heatmaps is applied only to the subset of genes and conditions selected by the user. Functional over-representation analyses for GO and KEGG are conducted using the clusterProfiler package (https://bioconductor.org/packages/release/bioc/html/clusterProfiler.html) (Yu *et al.* 2012), using gene–term mappings from Genome2D (Baerends *et al.* 2004). *P-*values are calculated using the hypergeometric test and adjusted for multiple testing using the Benjamini–Hochberg FDR.

### 2.6 Omics Integration

The Omics Integration module enables comparative analyses between chemical genomic and other omics datasets. Input matrices are aligned by shared gene identifiers. Pairwise associations are computed using the Spearman correlation coefficient (*ρ*). Overlap significance for user-defined thresholds is assessed with a two-sided Fisher’s exact test.

## 3 Results

**Figure 3:**
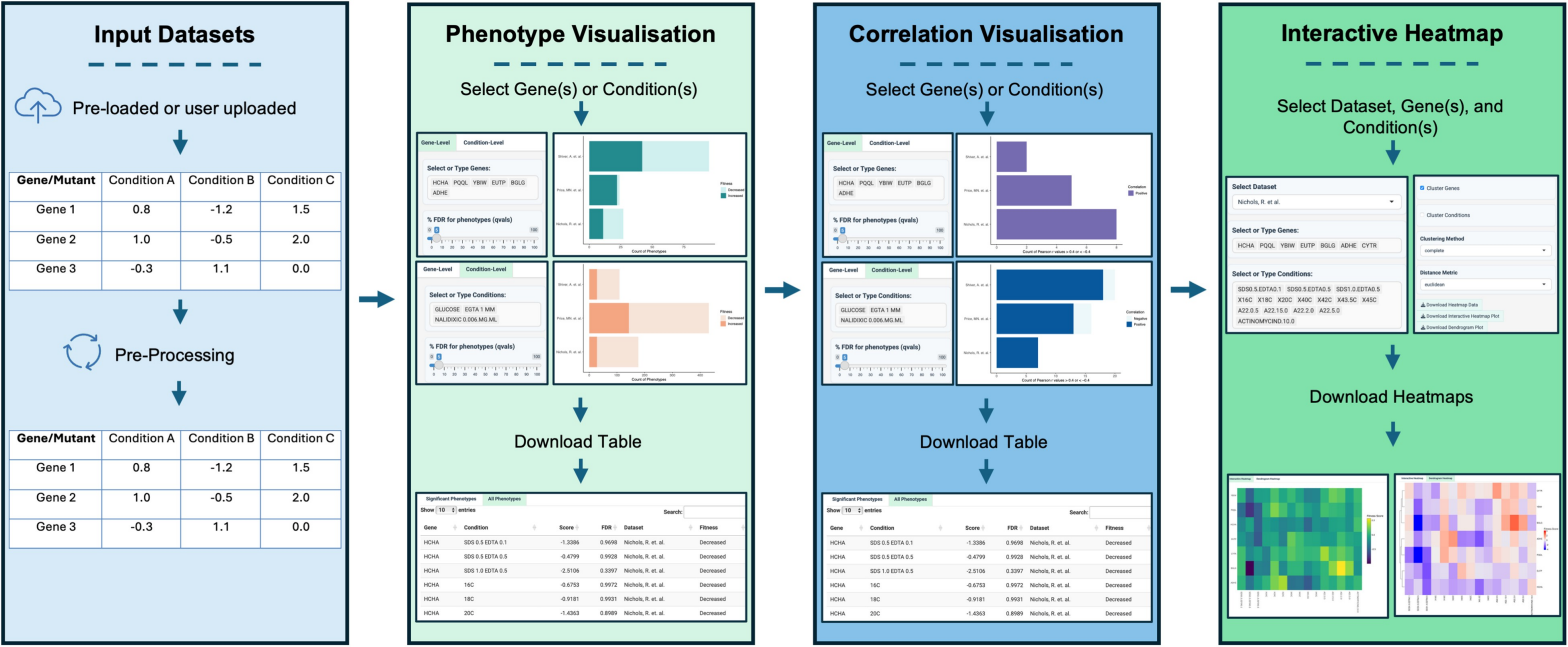
Basic functionality of ChemGenXplore. ChemGenXplore supports the visualization and analysis of chemical genomic datasets through four key features. Users can select pre-loaded datasets or upload their own in CSV format. Uploaded dataset is automatically pre-processed. The phenotype visualization allows users to explore fitness scores by selecting gene(s) or condition(s) of interest. The correlation visualization enables the identification of gene–gene and condition–condition correlations. The interactive heatmap facilitates the clustering of selected genes and conditions based on phenotypic profiles. Each feature supports interactive exploration and the option to download plots and tables for further analysis.

ChemGenXplore is designed to facilitate the interactive exploration of chemical genomic datasets, providing a flexible platform for analysing gene- and condition-specific phenotypes. Figure 3 presents a summary of the functionalities of ChemGenXplore. In the following sections, we outline these functionalities in detail.

### 3.1 Dataset upload

**Figure 4:**
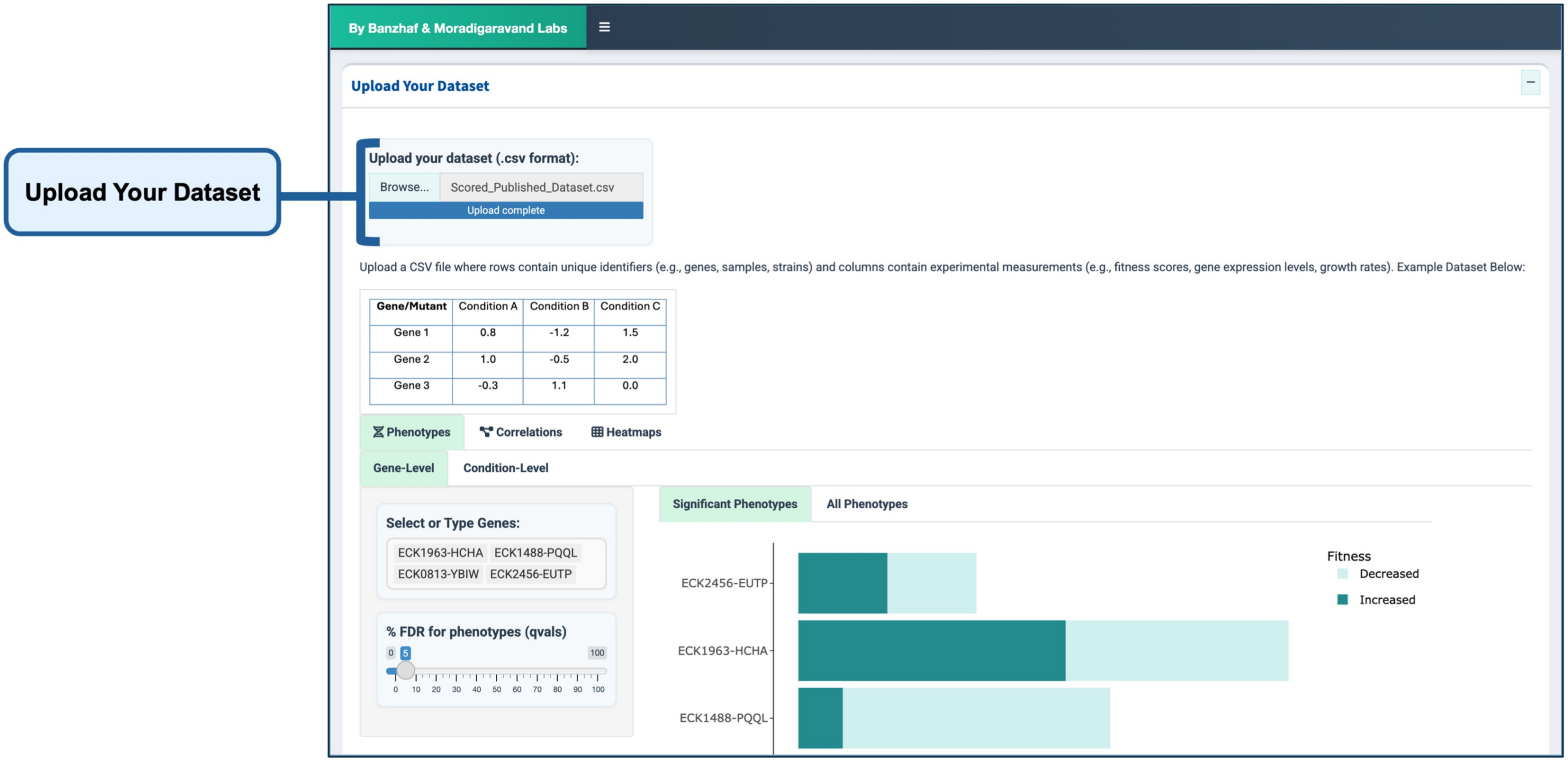
Dataset upload. Users can upload their datasets in CSV format, where rows represent genes/mutants and columns correspond to conditions. Once uploaded, the dataset is processed, allowing users to perform all available analyses, including phenotype visualization with FDR analysis, correlation analysis, and heatmap generation, following the same options as in the pre-implemented datasets.

ChemGenXplore provides users with the ability to upload their own datasets, where rows represent unique identifiers (e.g. genes, samples, strains) and columns contain experimental measurements (e.g. fitness scores, gene expression levels, growth rates) (Fig. 4). Once a dataset is uploaded, users can perform all the analyses available in the app, including phenotype visualization, correlation analysis, and heatmap generation. This feature makes it suitable for a wide range of chemical genomic datasets.

### 3.2 Phenotype visualization

**Figure 5:**
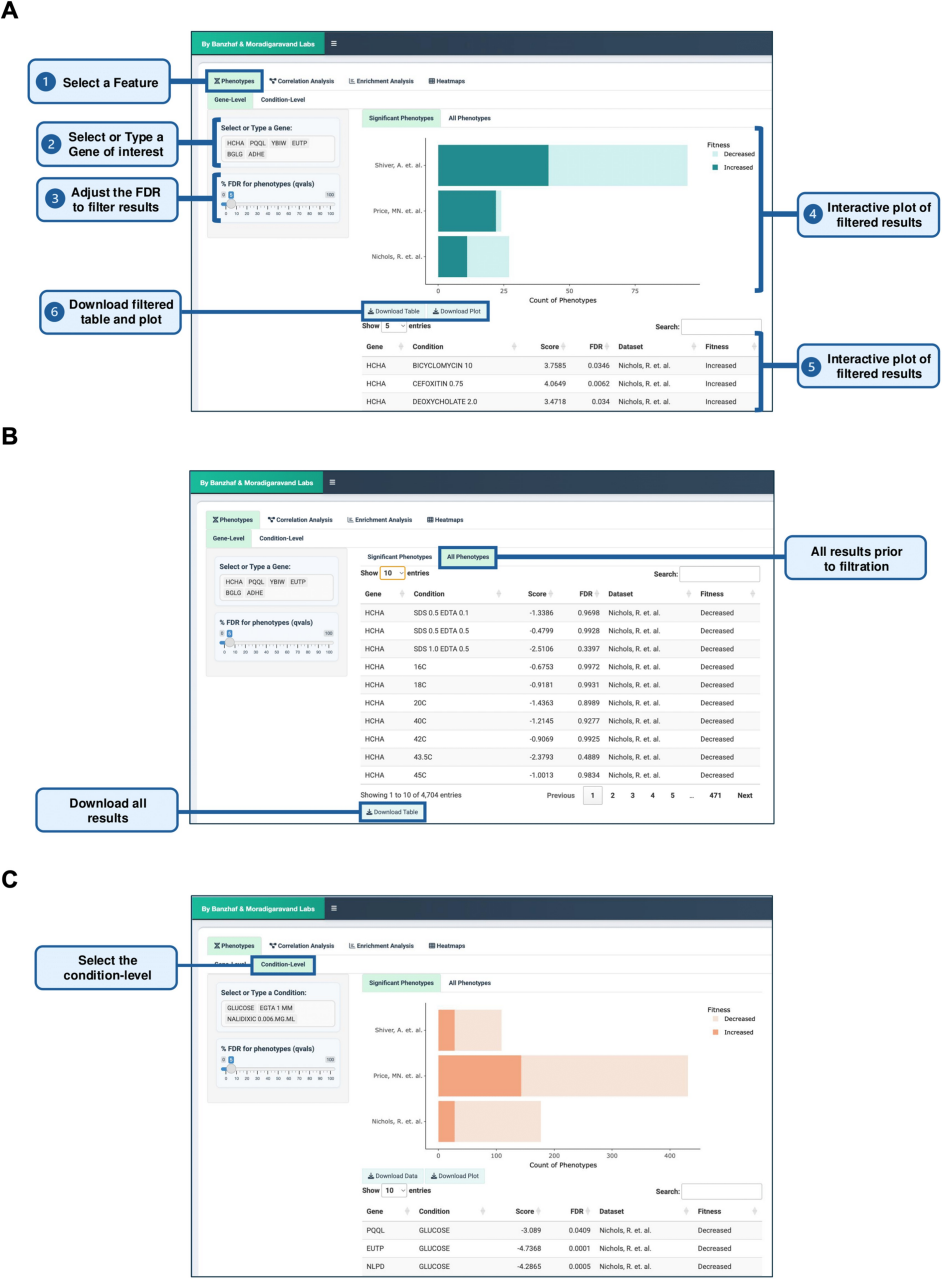
Phenotype visualization. (A) Users can select or type a single gene or multiple genes of interest to display phenotypes through interactive bar plots and data tables, with an FDR threshold to filter significant results. Both plots and tables are downloadable. (B) The “All Phenotypes” tab allows users to view all phenotypes related to the selected gene(s) without filtering. (C) Users can explore condition-specific phenotypes with the same display and download options.

The Phenotype Visualization feature enables users to explore gene- and condition-specific phenotypes through interactive bar plots and data tables. As shown in Fig. 5, users can select genes or conditions of interest and view corresponding phenotypic scores, with the option to filter results based on the FDR threshold. The FDR ensures statistical robustness by controlling false positives, reducing the likelihood of misleading associations that could mask true genotype–phenotype relationships. This feature is particularly useful for highlighting significant phenotypic changes and identifying potential gene–phenotype relationships.

### 3.3 Correlation analysis

Correlation analysis is a fundamental approach in chemical genomics for identifying functionally related genes and condition-specific phenotypic patterns. High correlation between phenotypic profiles is a strong predictor of functional connection between genes and can be used to characterize unannotated genes with known biological pathways (Nichols *et al.* 2011). Similarly, condition–condition correlations help identify clusters of chemical or environmental perturbations that produce comparable phenotypic effects, enabling researchers to group drugs with similar mechanisms of action or identify synergistic stress responses (Brochado *et al.* 2018).

**Figure 6:**
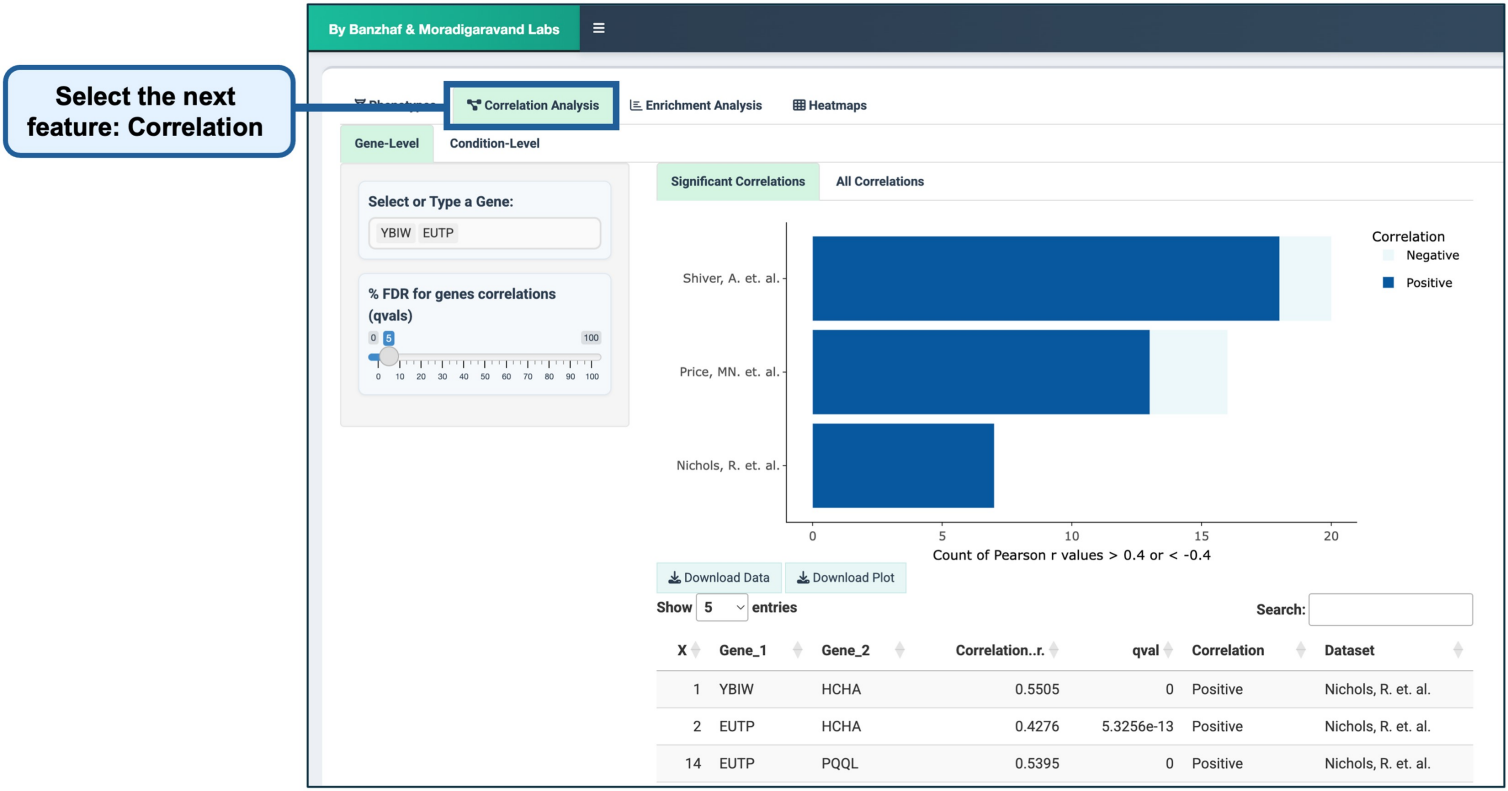
Correlation analysis. ChemGenXplore supports the visualization of gene–gene and condition–condition correlations using Pearson’s correlation coefficient. Interactive bar plots display correlations greater than ±0.4, with an FDR threshold to filter statistically significant correlations. Users can adjust the threshold and download results for further analysis as in the phenotype visualization tab.

The application supports the visualization of gene–gene and condition–condition correlations (Fig. 6). By using Pearson’s correlation coefficient, ChemGenXplore allows users to assess how gene fitness correlates across various conditions, as well as how conditions correlate based on their impact on phenotype. It provides interactive bar plots that display correlations greater than ±0.4. Users can adjust the FDR threshold to filter for statistically significant correlations and download the results for further analysis.

### 3.4 Enrichment analysis

**Figure 7:**
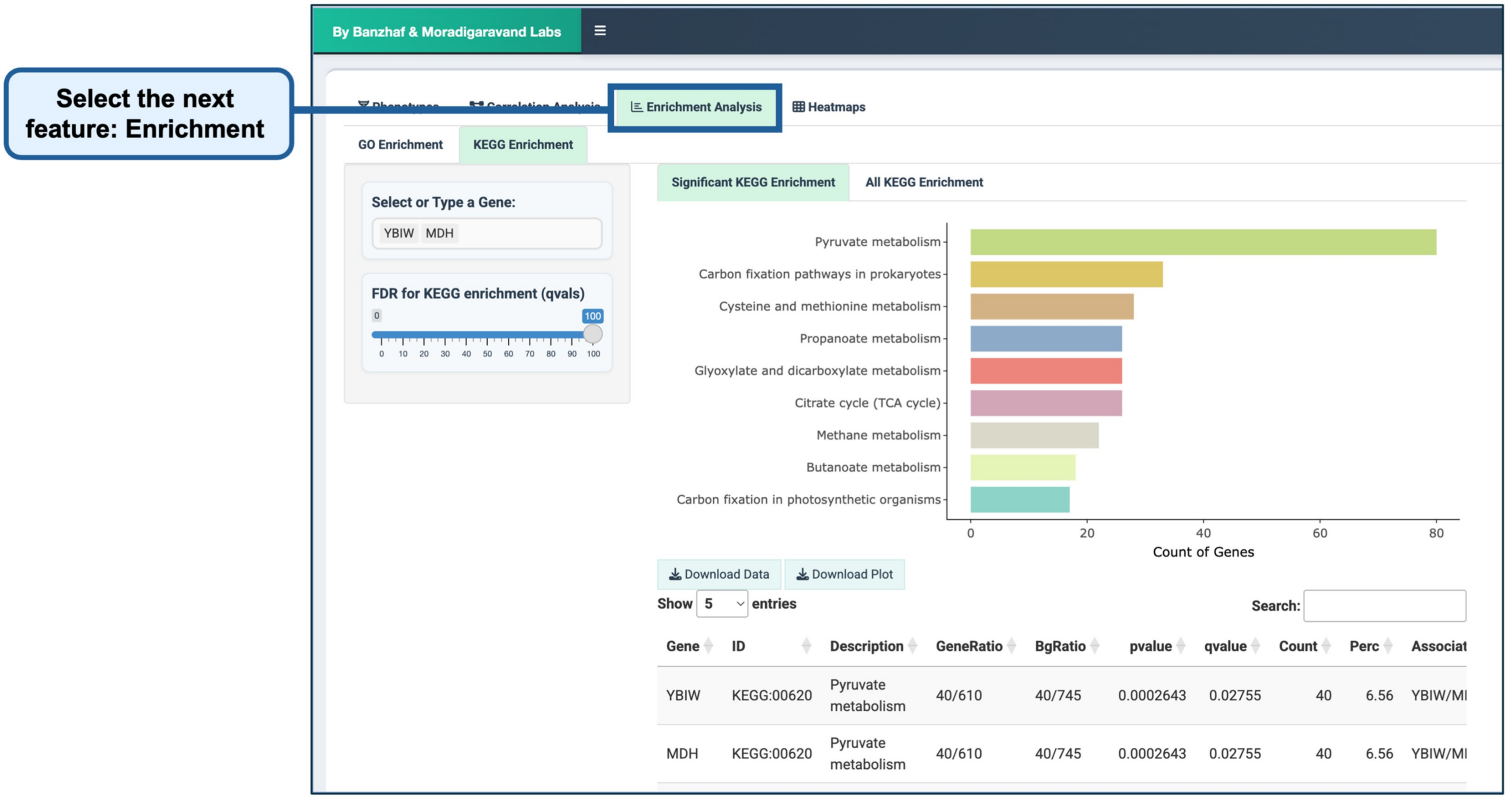
Enrichment analysis. Users can perform GO and KEGG enrichment analysis, following the same options as in the phenotype visualization and correlation analysis tabs.

Enrichment analysis identifies biological pathways and functional gene sets associated with phenotypic changes. By integrating GO and KEGG pathway enrichment analysis, ChemGenXplore enables researchers to uncover biological processes, molecular functions, and metabolic pathways enriched in their dataset. Enrichment results are visualized through interactive bar plots and tables, with the ability to filter results by FDR threshold (Fig. 7). This feature helps uncover biological processes and pathways linked to genes of interest. Users can download both the data and plots for further exploration.

### 3.5 Interactive heatmaps

**Figure 8:**
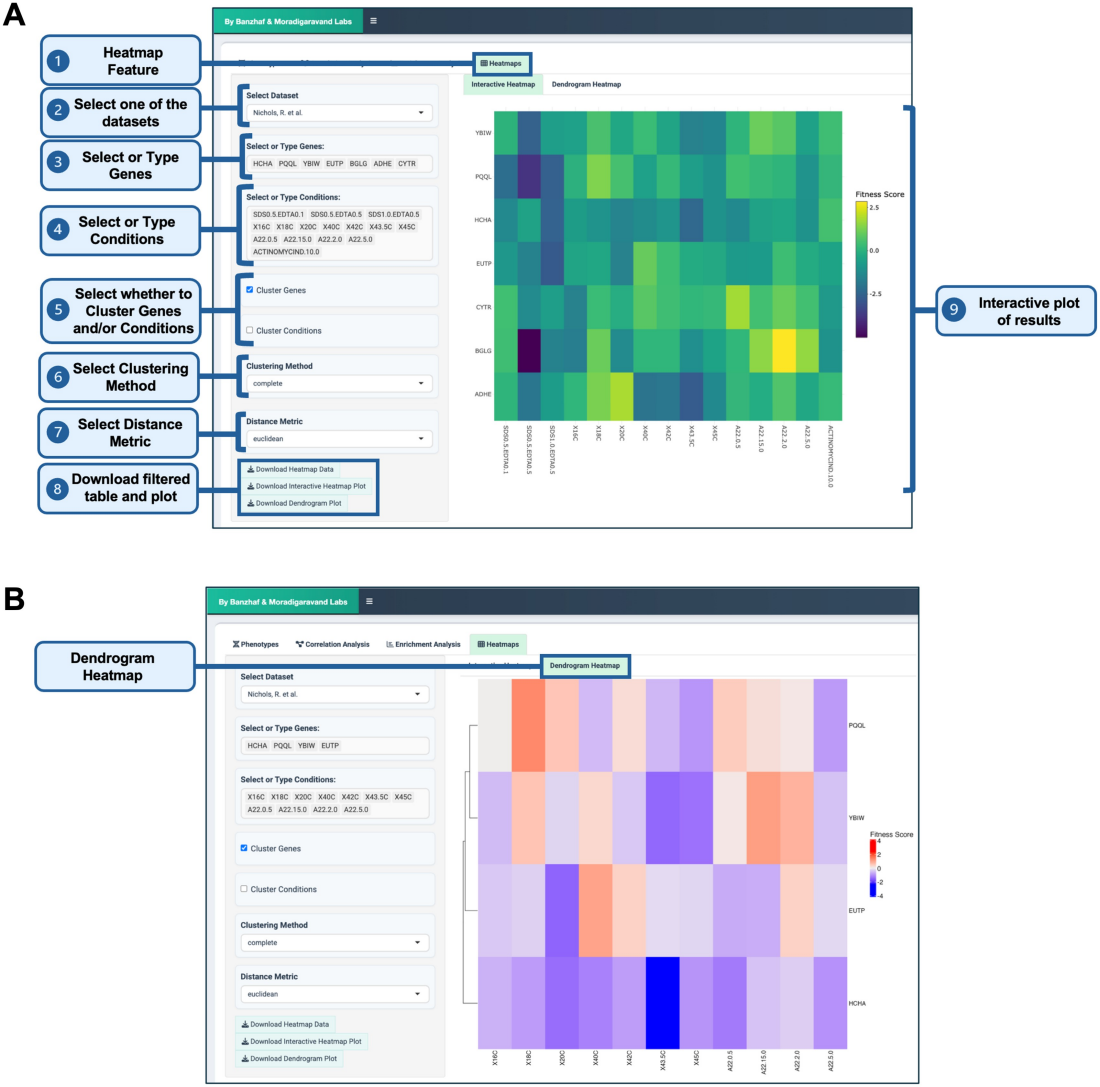
Interactive heatmaps. (A) Users can generate interactive heatmaps to visualize phenotypic relationships and patterns across conditions, applying various clustering methods and distance metrics. (B) Corresponding dendrograms display hierarchical relationships between genes and conditions. Both heatmaps and dendrograms are downloadable, following the same options as in the previous tabs.

Heatmaps are widely used in chemical genomics, enabling researchers to visualize phenotypic patterns. By clustering genes and conditions based on phenotypic profiles, heatmaps provide an intuitive way to detect functionally related genes. ChemGenXplore allows users to generate customizable, interactive heatmaps to visualize gene–condition fitness data (Fig. 8). Users can select genes and conditions of interest and apply clustering methods to group similar data points. Various clustering algorithms (e.g. complete, single, average, and ward) and distance metrics (e.g. Euclidean, Manhattan, Pearson/Spearman correlation, and cosine) are available, providing flexibility in data exploration. The heatmaps are fully interactive, allowing users to zoom in on specific areas and explore relationships between genes and conditions. Both the heatmap and dendrogram plots are available for download pdf and csv formats.

### 3.6 Omics integration

**Figure 9:**
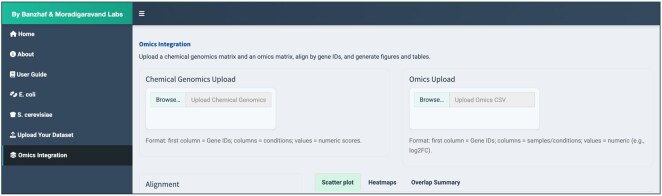
Omics integration. The omics integration tab enables comparative analysis of chemical genomic and other omics datasets. Users can upload chemical genomics and omics matrices, align them by shared gene identifiers, and access scatter plots, heatmaps, and overlap summaries.

To extend ChemGenXplore’s capabilities beyond chemical genomic data, an Omics Integration module was implemented to enable integrated analysis of chemical genomic and other omics datasets (e.g. transcriptomics, proteomics) (Fig. 9). This feature allows users to compare patterns in chemical genomic fitness profiles with corresponding molecular expression changes under similar conditions. Users can upload a chemical genomic matrix alongside a corresponding omics matrix, each structured with genes as rows and conditions as columns. The application aligns the datasets by shared gene identifiers and provides three analyses modes. The scatter plot analysis visualizes chemical genomic scores against omics values for each gene, reporting the Spearman correlation coefficient (*ρ*) and performing a Fisher’s exact test to assess enrichment of overlapping significant hits. The heatmaps display the two datasets side by side, allowing direct comparison of gene- and condition-specific patterns. The Overlap Summary quantifies shared and dataset-specific significant genes based on user-defined thresholds and visualizes the resulting categories. All plots and data tables can be downloaded. This functionality extends ChemGenXplore to support multi-omics data integration, enabling systems-level exploration of condition-specific phenotypes and broader biological responses.

### 3.7 Case study: visualizing antifolate stress responses

**Figure 10:**
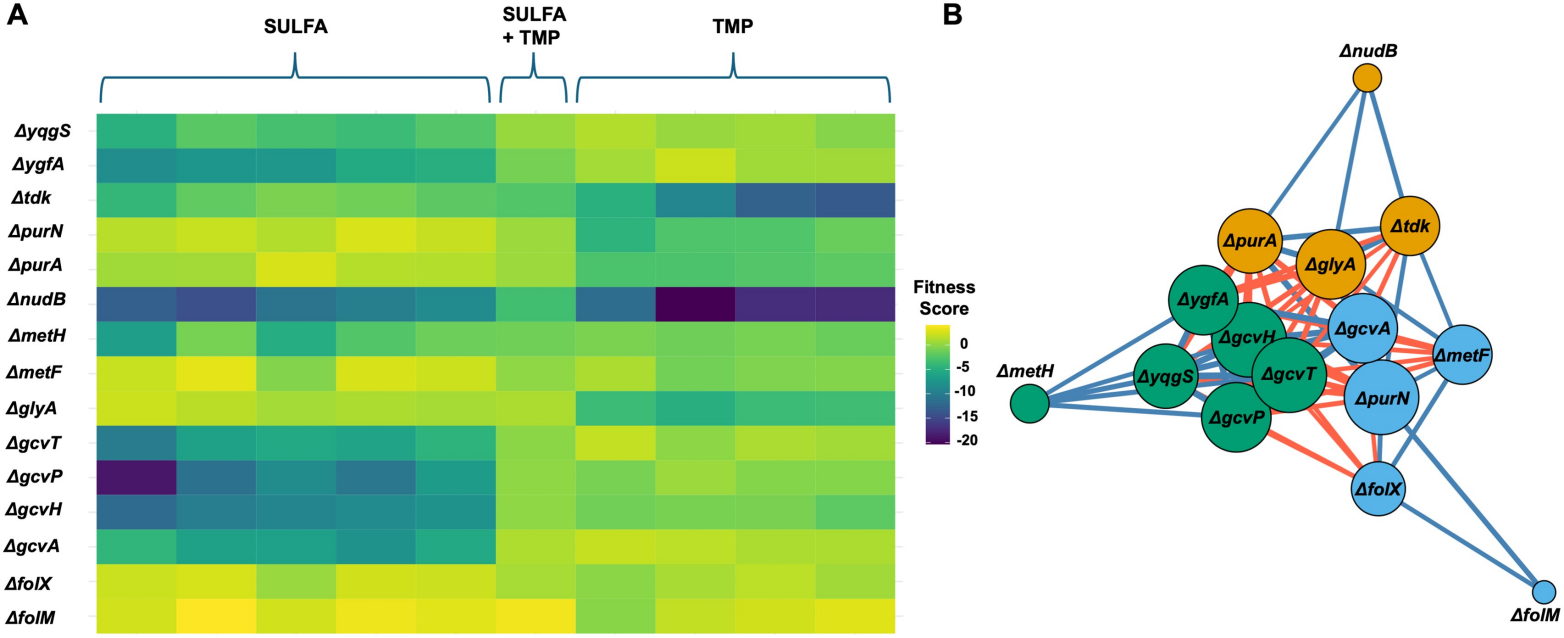
Differential antifolate responses revealed by ChemGenXplore. (A) A heatmap visualizing phenotypic profiles of mutants involved in tetrahydrofolate (THF) and one-carbon metabolism under sulfonamide, trimethoprim (TMP), and combination stress. Clustering reveals distinct responses, including sulfa-specific sensitivity in Δgcv and ΔygfA, TMP-specific sensitivity in ΔglyA, hypersensitivity in ΔnudB, and increased resistance in ΔfolM and ΔfolX. (B) Gene–gene correlation network based on Pearson correlation of fitness profiles across antifolate conditions (|*r*| > 0.6). Node colours correspond to Louvain clusters.

To demonstrate ChemGenXplore’s utility in exploring phenotypic patterns across experimental conditions, we selected a subset of genes involved in tetrahydrofolate (THF) and one-carbon metabolism under antifolate stress. This example replicates a key finding from Nichols *et al.* (2011), which characterized gene-specific responses to sulphonamides, trimethoprim (TMP), and their combination. The resulting heatmap in Fig. 10A reveals that the fitness response of ΔgcvA, ΔgcvP, ΔgcvT, ΔgcvH, and ΔygfA mutants was selectively reduced under sulphonamides, while ΔglyA was sensitive to TMP. ΔnudB deletion conferred hypersensitivity to all folate stresses, consistent with its role in folate biosynthesis. In contrast, ΔfolM and ΔfolX mutants exhibited increased resistance across conditions. These results are consistent with Nichols *et al.* (2010). Furthermore, we generated a gene–gene correlation network based on Pearson correlations of fitness profiles across antifolate conditions. As shown in Fig. 10B, the network reveals clusters of genes with similar responses to antifolate stress. Edge colours indicate the sign of the correlation (positive or negative), while node colours correspond to gene clusters identified using the Louvain algorithm, a modularity-based method for detecting communities within networks (Blondel *et al.* 2008). This combined visualization illustrates ChemGenXplore’s ability to uncover similar gene responses and functionally related gene groups under stresses. This highlights ChemGenXplore’s role as a hypothesis-generation and data-exploration platform for visualizing phenotypic patterns and functional gene relationships in chemical genomic datasets.

## 4 Discussion

The first release of ChemGenXplore provides a pre-implemented *E. coli* and *S. cerevisiae* datasets alongside the option for users to upload their own datasets. The platform also incorporates an omics integration module that enables comparative analysis between chemical genomic and other omics datasets. In future versions, we aim to expand its functionality by integrating additional species from ongoing and future chemical genomic screens. By consolidating chemical genomic data from various species, ChemGenXplore serves as a centralized tool for the chemical genomics community, facilitating comparative analyses to identify conserved and species-specific phenotypic responses. A key advantage of this approach is its ability to address a wide range of research questions spanning multiple fields, including gene–function mapping, genetic and drug interactions, and mode-of-action studies for drug discovery. By supporting data sharing, reproducibility, and collaboration, ChemGenXplore will continue to integrate additional datasets and enhance its functionality. We look forward to a growing community of users and contributors to support ongoing research in the field.

## Data Availability

The data underlying this article are available in *Zenodo*, at https://doi.org/10.5281/zenodo.16753661. The datasets were derived from sources in the public domain: Nichols *et al.* (2011), Price *et al.* (2018), Shiver *et al.* (2017), and Viéitez *et al.* (2022).
